# A systematic review and narrative synthesis of health literacy interventions among Spanish speaking populations in the United States

**DOI:** 10.1186/s12889-024-19166-6

**Published:** 2024-06-27

**Authors:** Joel Hernandez, Liliana Demiranda, Priyanka Perisetla, Lauren Andrews, Keer Zhang, Rebecca Henderson, Ajay Mittal, Hannah F. Norton, Melanie G. Hagen

**Affiliations:** 1 Equal Access Research, College of Medicine, University of Florida, 1549 Gale Lemerand Drive, 4 Floor, Suite 4592, Gainesville, FL 32610-3008 USA; 2 Department of Internal Medicine, College of Medicine, University of Florida, Gainesville, FL USA; 3 University of Florida Health Science Center Libraries, Gainesville, FL USA; 4 University of Central Florida College of Medicine, University of Central Florida, Orlando, USA; 5 David Geffen School of Medicine, University of California Los Angeles, Los Angeles, USA; 6 College of Medicine, University of Florida, Gainesville, Florida, USA

**Keywords:** Limited English Proficiency (LEP), Health literacy, Spanish speaking, Intervention, Systematic review

## Abstract

**Background:**

While many populations struggle with health literacy, those who speak Spanish preferentially or exclusively, including Hispanic, immigrant, or migrant populations, may face particular barriers, as they navigate a predominantly English-language healthcare system. This population also faces greater morbidity and mortality from treatable chronic diseases, such as hypertension and diabetes. The aim of this systematic review was to describe existing health literacy interventions for patients with a Spanish-language preference and present their effectiveness.

**Methods:**

We carried out a systematic review where Web of Science, EMBASE, and PubMed were queried using MeSH terms to identify relevant literature. Included articles described patients with a Spanish-language preference participating in interventions to improve health literacy levels in the United States. Screening and data abstraction were conducted independently and in pairs. Risk of bias assessments were conducted using validated appraisal tools.

**Results:**

A total of 2823 studies were identified, of which 62 met our eligibility criteria. The studies took place in a variety of community and clinical settings and used varied tools for measuring health literacy. Of the interventions, 28 consisted of in-person education and 27 implemented multimedia education, with 89% of studies in each category finding significant results. The remaining seven studies featured multimodal interventions, all of which achieved significant results.

**Conclusion:**

Successful strategies included the addition of liaison roles, such as *promotores* (Hispanic community health workers), and the use of multimedia *fotonovelas* (photo comics) with linguistic and cultural adaptations. In some cases, the external validity of the results was limited. Improving low health literacy in patients with a Spanish-language preference, a population with existing barriers to high quality of care, may help them better navigate health infrastructure and make informed decisions regarding their health.

**Registration:**

PROSPERO (available at https://www.crd.york.ac.uk/prospero/display_record.php?ID=CRD42021257655.t).

**Supplementary Information:**

The online version contains supplementary material available at 10.1186/s12889-024-19166-6.

## Introduction

While health literacy (HL) is a multifaceted concept [1, 2] almost all definitions relate HL to “the literacy and numeracy skills that enable individuals to obtain, understand, appraise, and use information to make decisions and take actions that will have an impact on health status” [3]. Low HL has been linked to poorer health outcomes, including increased mortality [4, 5]. HL has increasingly been recognized as a potentially important factor mediating health disparities, especially those related to race and ethnicity [5], and has been suggested as an important mediator of the relationship between socioeconomic status and health [6]. This may be due to communication barriers with physicians and difficulty understanding and making use of medical resources [5].

As a concept, HL has sometimes been poorly defined. A recent systematic review which sought to clarify the concept found that scholars commonly characterized HL along three main domains: knowledge of health/healthcare systems, processing and using information related to health and healthcare, and the ability to maintain health through collaboration with health providers [7]. Other theoretical frameworks developed for HL understand the concept through its effects. For example Nutbeam established a useful framework for understanding the benefits of health literacy through a “health outcomes model” in which HL is comprised of functional HL, the basic skills necessary for everyday health functioning, communicative/interactive HL, the more advanced skills needed to act independently with “motivation and self-confidence,” and critical HL, the ability to analyze and use information to “exert greater control over life events and situations” allowing people to respond adversity and to advocate for themselves [8, 9]. HL is sometimes understood as not only a skill, but an important social determinant of health, with community level and public health implications [10].

While many U.S. residents struggle with limited health literacy, there may be a particular barrier among those who speak Spanish preferentially or exclusively, including Hispanic, immigrant or migrant populations. In the United States, minority groups, immigrants, migrants, and nonnative English speakers have lower health literacy scores than White adults and are at higher risk of having poor HL, making them more susceptible to the adverse outcomes associated with low HL [11]. Hispanics are the largest group of nonnative English speakers and preferential Spanish speakers in the U.S. and have low rates of HL compared to other populations [5]. Limited English proficiency may be a factor that contributes to poorer health outcomes and reduced quality of care, especially in a predominantly English language-based health care system with a shortage of bilingual and culturally competent providers [12]. For example, one recent study found higher rates of obesity among Spanish speakers in the United States [13]. These factors, in combination with a lack of healthcare access and insurance coverage, may contribute to higher morbidity and mortality rates among Hispanics due to chronic diseases such as diabetes and obesity [14].

Methods to accommodate the HL needs of patients with a Spanish-language preference (SLP) may therefore be important in improving health equity [15]. While strides have been made in community-based educational efforts and the translation or cultural adaptation of health communication tools and processes [16], there are limited data on effective interventions to improve HL for patients with SLP in the United States [17]. The literature on interventions targeting HL in the United States has frequently grouped together populations of immigrants who do not share a common language [18] or, conversely, focused only on individuals from a single nationality [19, 20]. Given the gap in the literature synthesizing research on HL interventions for patients with SLP in the United States and the important association between HL and health outcomes, we conducted a systematic review of the literature that summarizes and evaluates the effectiveness of HL intervention strategies for patients with SLP in the United States. The aim of this systematic review was to describe existing HL interventions for patients with SLP and present their reported effectiveness.

### Methods

#### Protocol and registration

The protocol for this review was registered with PROSPERO (CRD42021257655). The use of the Preferred Reporting Items for Systematic Reviews and Meta-Analysis (PRISMA) enabled authors to follow best practices in conducting the review [21].

#### Search strategy and screening

**Fig. 1:**
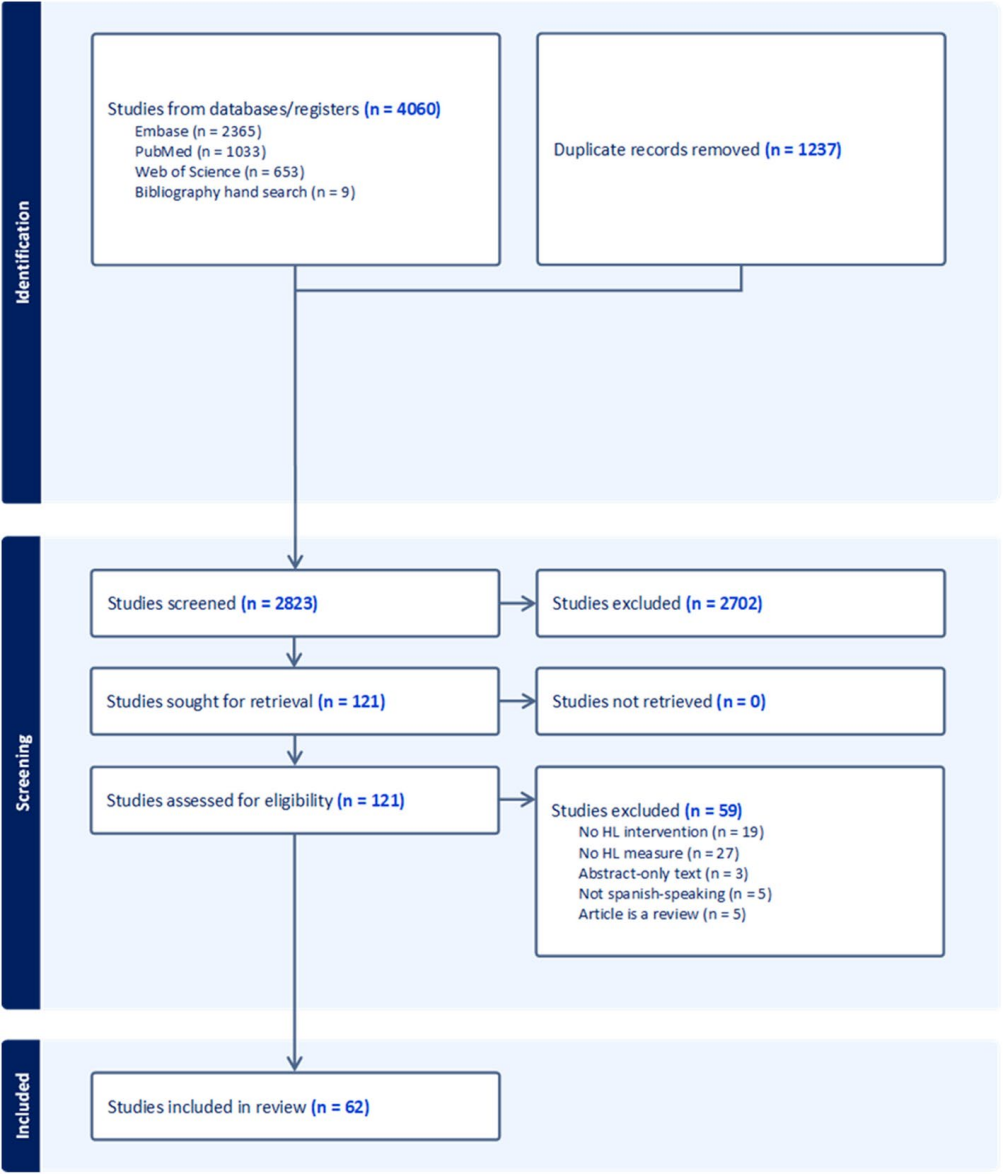
PRISMA flow diagram for studies considered for the systematic review

Searches were conducted in the PubMed, MEDLINE, Web of Science, and Embase databases and data was extracted from these databases between January 20, 2020 and April 27, 2023 (Fig. 1.). The keywords for each database included: “health literacy” and “intervention” or “Spanish”, “Hispanic” or “LEP,” or “limited English proficiency.” Databases were queried to include only articles published between January 1, 2011 and April 27, 2023. In 2010, the U.S. The Department of Health and Human Services unveiled the National Action Plan to Improve Health Literacy, bringing more attention to this matter and inspiring more research on HL. Our review also avoids redundancy with a 2011 comprehensive review [5], which found no interventions focused on HL in Spanish-speaking populations, with only three mentioned measures of HL in this population.

After removing duplicates, two reviewers, P.P. and L.D., independently reviewed titles and abstracts to select potentially eligible articles based on the inclusion/exclusion criteria described below. Any disagreements regarding the inclusion of a study were resolved by a third reviewer, J.H. Bibliographies of included studies were subsequently hand searched.

#### Inclusion & exclusion criteria

Inclusion criteria for this literature review included articles that a) featured participants with SLP, b) described interventions that occurred in the United States, c) described interventions that were designed to mitigate the effects of low HL in participants with SLP and improve the use of health services or the health outcomes in these populations, d) were shared in an online format in indexed scientific journals, e) were written in English or Spanish, f) were published in 2011–2023, g) were randomized control trials (RCTs), pre/post (PP) studies, prospective cohort (PC) studies, cross-sectional (CC) studies, or mixed methods studies and h) measured effectiveness of intervention using HL assessment tools or health outcomes.

Exclusion criteria included studies of outcomes related to numeracy or literacy alone without reference to HL because such interventions were found to differ from those that dealt with these issues in the context of HL. We also excluded studies that did not report HL interventions targeting Spanish-speakers in the United States.

#### Assessment of methodology quality

We assessed the methodological quality of each included study using the Revised Tool to Assess Risk of Bias in Randomized Trials (RoB 2) [22] and the Risk Of Bias In Non-randomized Studies—of Interventions (ROBINS-I) tool for assessing risk of bias in the different interventions analyzed (RCTs, PP studies, PC studies, CC studies, and mixed methods studies) [23]. Two review authors (P.P. and L.D.) independently performed quality assessments. Disagreements regarding the overall assessments were resolved through discussion, with a third reviewer as the final arbitrator (J.H.). Bibliographies of included studies were subsequently hand searched.

#### Data synthesis

After piloting, four reviewers (J.H., L.A., P.P., L.D.) conducted data extraction using a standardized data extraction template (Appendix 1). Due to the heterogeneity of interventions, outcomes assessed, and varying durations of interventions, we did not pool the data and instead conducted a narrative analysis. We conducted a thematic analysis of identified studies and grouped studies for synthesis on the basis of identified categories. This process consisted of iterative discussions of the studies by all members of the study team and was based on published guidelines for Synthesis without Metanalysis (SWiM) [24]. Our data synthesis specifically grouped studies based on the categories of study characteristics, measures of effectiveness, reported effectiveness by intervention type, and quality assessment. We stratified the results by intervention type. While we did not focus on migrant status specifically, this could be estimated by one of our data extraction items, country of origin.

## Results

### Study characteristics

After removal of duplicates, 2,823 titles and abstracts were screened for inclusion using the criteria described above. A manual search of bibliographies yielded eight additional articles for screening. A total of 121 potentially relevant articles were selected using the inclusion criteria described above. After a detailed full-text analysis of each study, 62 studies were included, and 59 were excluded, as indicated in Fig. 1. This included 17 RCTs, 35 PP studies, 3 PC studies, 3 CC studies, and 4 mixed methods studies. A summary of the study characteristics can be found in Table 1, 2, and 3. The studies encompassed mainly female, middle-aged adults (range: 30 to 50); only two studies included participants under the age of 18 [25, 26] and no studies were focused solely on pediatric populations. Only a minority of participants had graduated from college. Sample sizes varied from 10 to 943. Interventions included in-person education (*n* = 28), multimedia education (*n* = 27) and other types of multimodal strategies (*n* = 7). Eighteen studies made use of lay health advisors and *promotores*.

**Table 1:** Summary of the study characteristics-in person education

In-Person Education								
**Study**	Intervention type	Intervention description	Theoretical framework	Study design	Target population	Measure of effectiveness	Primary outcomes	Study setting
**Auger 2015 **[15]	In-person Education	Multimodal intervention: use of *fotonovelas* as an educational tool, health education facilitation by the teacher and lay health educator	Relational-cultural theory and the Stages of change model	Nonrandomized mixed-strategy	Low-income pregnant Latinas	Prenatal knowledge surveys developed by the researchers	Increased knowledge of pregnancy, childbirth, and breastfeeding (*p* < .001) and confidence in navigating pregnancy, caring for oneself and the baby, and interacting with health professionals (*p* ≤ .05). No significant change in perceived social support	Community health center and local health department, Central North Carolina
**Buckley 2015 **[27]	In-person Education	"Social club" hosted by *navegantes* for 2 h every week over 5 weeks for participants to share their stories, participate in fun activities, and learn about the basic nutrition and metabolic syndrome risk factors	Social cognitive theory	Pretest–Posttest Design	Open to all interested individuals	Measured changes in health outcomes between the baseline and eight-week time point for individuals who participated in at least six out of the eight sessions	88.9% of 126 participants increased health literacy and over 60% decreased at least one risk factor associated with metabolic syndrome. Change for those that improved, [mean (SD)]: Weight [− 6.0 lbs (5.2)]; BMI [− 1.1 (1.0)]; Waist Circumference [− 2.2 inches (1.5)]; Blood Glucose [− 26.3 mg/dl (27.5)]; LDL Cholesterol [− 19.1 mg/dl (16.8)]; Systolic BP [− 11.1 mmHg (9.5)]; Health Literacy Test (*n* = 117) [+ 22.2% (19.7%)]	Free clinic in Providence, Rhode Island
**Castañeda 2016 **[28]	In-person Education	6-week, culturally tailored, *promotora*-based group instruction including healthy diet instruction, assistance tracking preventive service visits, and follow-up calls	RE-AIM (reach, effectiveness, adoption, implementation, and maintenance) framework	Pretest–Posttest Design	Spanish-speaking Mexican-born women	Chew Health Literacy Screener and Newest Vital Sign (NVS)	Participants improved their self-reported cancer screening, BC knowledge (Mpre = 2.64, Mpost = 3.02), daily fruit and vegetable intake, and ability to read a nutrition label (*p* < 0.05). No significant change in measures of BC cultural beliefs, health literacy, and screening intentions	San Ysidro Health Center. San Diego, California
**Chen 2022 **[29]	In-person Edudcation	Community health workers-led a culturally appropriate education program in-clinic and home visits	None	Randomized Control Trial	Spanish-speaking caregivers of Latin American pediatric patients with atopic dermatitis	Measured primary outcome was measured by measuring adherence rate of emollient (white petrolatum) application	Not statistically significant increased adherence rate in the *promotora* group versus *nonpromotora* group for weeks 1–12 (median [IQR] 43% [26%–61%] vs. 20% [11%–49%], *p* = .09) and for weeks 5–12 (median [IQR] 46% (22%–57%) versus 17% [7%–41%], *p* = .06). Increased AD knowledge (median [IQR]: 0.830 [0.810–0.880]) relative to the *nonpromotora* group (median [IQR]: 0.770 [0.750–0.833], *p* = .06)	Pediatric dermatology clinic at hospital
**Cruz 2013 **[30]	In-person Education	90 min training session conducted by promoters focusing on general knowledge about diabetes, risk factors, and prevention and control of diabetes	None	Pretest–Posttest Design	Hispanic seniors with or without diabetes	Questionnaire developed by researchers	Significant improvement on diabetes knowledge for diabetic participants comparing pre- and posttest scores (13.7 vs. 18.6, *p* < 0.001; Cohen’s d = 1.2), and for nondiabetic participants (12.9 vs. 18.2, *p* < 0.001; Cohen’s d = 1.2). For health promoters it also increased significantly (13.1 vs. 17.8, *p* < 0.001; Cohen’s d = 36)	Senior centers in California, Texas, and Washington DC
**Esquivel 2014 **[31]	In Person Education	Individualized education provided over 3 months on high salt foods, when to call the physician, when to report weight gain and the use of diuretics	None	Randomized Control Trial	Hispanic adults with HF	Short assessment of health literacy for Spanish-speaking adults (SAHLSA-50)	Self-care management scores significantly improved from a mean of 49 to 81 (*p* = 0.02) and teach-back scores significantly improved from a mean of 3.6 to 4.0 (*p* = 0.04) for intervention group. Improvements in depression and anxiety scores were present but not significant	San Francisco General Hospital. San Francisco, CA
**Han 2018 **[32]	In-person Education	Four weekly hypertension education sessions followed by monthly phone counseling and optional text messaging	None	Pretest–Posttest Design	Spanish-speaking adults ≥ 18 years old,	Used several established HL assessment tools and evaluated participants’ blood pressure	10 of 11 participants achieved BP control (< 140/90 mm Hg) at 16 weeks. The effect sizes of health literacy and psychosocial variables ranged from 0.1 to 1.7 in absolute value	Baltimore, MD
**Jandorf 2012 **[33]	In-person Education	Education program where trained peer volunteers (breast and cervical cancer survivors or women living with diabetes) share their personal stories and educate about breast and cervical cancers and the recommended screening tests	None	Pretest–Posttest Design	Immigrant Latina women and their partners in New York and Arkansas	Questionnaire developed by researchers	Significant improvements in cancer knowledge scores across all sites (M = 22.70, SD = 23.85, *p* < .001). No significant across-site differences for breast self-exam or Pap test screening adherence (21.4% and 74.0%, respectively, for most participants)	Community-based and faith-based organizations or private home
**Kaphingst 2011 **[34]	In-person Education	One-hour educational session, with activities delivered by LHAs using the culturally tailored flip chart, role playing activities, and extensive small-group discussions regarding family health history (FHH)	None	Pretest–Posttest Design	Spanish-speaking adults ≥ 18 years old	Questionnaire developed by researchers	Significantly greater mean change intentions to discuss FHH with family members in the intervention group compared to control (0.77[SD = 1.4] vs 0.42[SD = 1.1], *p* = 0.003) but not to discuss with doctors (*p* = 0.11). Significantly greater mean change in self-efficacy to discuss FHH with family members and doctors (0.66[SD = 1.4] vs 0.24[SD = 1.0], *p* = 0.0003 and 0.69[SD = 1.3] vs 0.30[SD = 1.1], *p* = 0.0008; respectively. No significant difference in posttest mean knowledge score	2 community health centers in Oakland, CA and Washington, DC
**Laughman 2017 **[35]	In-person Education	Culturally sensitive health education classes hosted by certified bilingual Latina health educator regarding breast cancer and mammography	None	Pretest–Posttest Design	Underserved adult female Latinas in North Carolina without history of breast cancer	Questionnaire developed by researchers	Prepost results varied depending on the three major themes: risk factors, screening and signs and symptoms. Of the 25 total questions, 15 (60%) showed statistically significant improvement in correct response rate, 8 showed nonstatistically significant improvement, and no question showed statistically significant worsening. The study was not powered statistically to show differential impact due to the educational setting	Home and church in rural and urban settings
**Martin 2016 **[36]	In Person Education	Two separate half-day highly standardized and interactive educational symposia designed to address the activation domains of care, comparing Spanish speaking (SP) and English speaking (ES) participants	None	Pretest–Posttest Design	8–21-year-old patients with Crohn’s disease or ulcerative colitis	Patient activation measure (PAM)	Statistically significant increase in patient activation measure (mean pretest score was 62.9 [SD 12.5], mean posttest score was 69.4 [SD 15.1]; *p* < 0.001). SP-patients showing the largest incremental change among the groups, although only the ES-parents group had sufficient sample size (*n* = 26) to achieve statistical significance	Stanford Children’s IBD Center
**Mas 2017 **[37]	In Person Education	GED curriculum supplemented with disease-specific and health-care-related information delivered over five two-hour sessions	None	Pretest–Posttest Design	Adults ≥ 21 years old fluent in Spanish	S-TOFHLA	No statistically significant score changes between pre- and posttest across groups. Only participants in the “marginal functional health literacy” and “adequate functional health literacy” categories saw a statistically significant gain between pretest and posttest	U.S.-Mexico border community college and public university, NM
**Mas 2018 **[38]	In-person Education	Community-based cardiovascular (CVD) education and promotion	Theories of health literacy and health behavior, sociocultural approaches to literacy and communication, and adult learning theory	Pretest–Posttest Design	Spanish-speaking adults ≥ 21 years old with low-to-intermediate English proficiency	Adapted version of Spanish *Cuestionario de Salud Cardiovascular* (CSC) (Cardiovascular Health Questionnaire) Test of Functional Health Literacy in Adults (TOFHLA)	Significant increase for the intervention group compared to control group regarding change in cardiovascular knowledge (2.3, 0.01–4.7, *p* = 0.049), TOFHLA (5.0, 1.2–8.8, *p* = 0.011), and numeracy scores (1.0, 0.1–2.0, *p* = 0.037)	El Paso, TX
**Mojica 2016 **[39]	In Person Education	Education-plus-navigation intervention for breast, cervical, and colorectal cancer based on the Health Belief Model	Health belief model	Pretest–Posttest Design	Latinas who had never had a mammogram, Pap test, or stool blood test	Self-reported receipt of a mammogram, Pap test, or stool blood test	Statistically significant improvement in 3/5 questions of the knowledge, attitudes, and beliefs survey regarding cancer (*p* < 0.001), and in all questions regarding beliefs/attitudes related to early detection (*p* < 0.001)	San Antonio, Texas
**Moralez 2012 **[40]	In-person Education	*Promotoras* led and home-based educational intervention used culturally appropriate guided group discussion to reduce barriers and increase access to screenings	None	Pretest–Posttest Design	Hispanics who are poor or live in underserved rural and border communities of Yakima Valley, Washington	Questionnaire developed by researchers and rates of changes in Screening Awareness and Practices	Significant decrease in the proportion of participants agreeing with the statement “there is nothing that can be done to prevent cancer” form baseline to follow-up (46.7%-18.3%, p = 0.003). No significant changes with the beliefs “A tumor is can always cancerous” and “Finding cancer early helps you survive longer” with the latter being 98.3% at baseline. Significant increase in Colorectal Cancer Screening Awareness and Practices at follow-up, with varying scores depending on the specific practice	Home of community members
**Nitsos 2017 **[41]	In Person Education	Bilingual and bicultural research assistants explained parents the importance of Tummy Time (TT), demonstrated how to carry it out TT, and provided a TT brochure	None	Pretest–Posttest Design	Spanish-speaking Latino parents, expectant parents, or caregivers of infants	Questionnaire developed by researchers	Pre/posttest data indicated an increase in knowledge (z = − 2.03, *p* = 0.04), especially in areas like when to start and how often to implement Tummy Time	Church and obstetric clinic; West Columbia and Lancaster, South Carolina
**Ockene 2012 **[42]	In Person Education	Usual care (UC) vs lifestyle intervention care (IC) in which individual (3) and group sessions (13) over 12 months to improve eating habits and increase daily steps using principles of social cognitive theory and patient-centered counseling	Social cognitive theory and Patient centered counseling	Randomized Controlled Trial	Low-income, Spanish-speaking Latinos at high risk for diabetes	Health outcomes	The IC group lost significantly more weight (− 2.5 lb; *p* = .004) and had a more significant reduction in HbA1c (− 0.07; *p* = .009) and BMI (− 0.46; *p* = .004). No statistically significant intervention effect on fasting blood glucose, insulin, leisure-time physical activity, or depression score	Greater Lawrence Family Health Center. Lawrence, Massachusetts
**Otilingam 2015 **[43]	In-person Education	Workshops using cooking demonstrations, *fotonovelas*, experience sharing, and game show formats to reduce dietary fat barriers, build dietary fat self-efficacy, and provide cues to action. Workshops focused only on dietary fat and heart health, a waitlist control group, and a posttest only control group	None	Randomized Control Trial	Latina women ≥ 40 years old	NVS Behaviors to reduce dietary fat were measured with the Fat-Related Diet Habits Questionnaire	No statistically significant difference in change between the heart plus brain and the heart only interventions. However, when contrasting interventions groups together to control, there was a significant improvement at follow-up (*p* = .0036) but not at posttest (*p* = .1813) in behaviors to decrease fat consumption	Community clinic. Los Angeles, CA
**Peña-Purcell 2014 **[44]	In Person Education	Culturally sensitive, empowerment-based, diabetes self-management education program	Social cognitive and Self-regulation theories	Prospective, quasiexperimental, repeated-measure design	Hispanic adults ≥ 40 years old, diagnosed with type 2 diabetes	Spoken Knowledge in Low Literacy in Diabetes (SKILLD) Health outcomes	Significant increase in diabetes knowledge (median baseline score was 6/10 for both groups, changed to 7 in intervention and 5 in control group, *p* < .01) No significant relationship was found for any item in the scale other than Item 7 (normal fasting blood glucose), from those in the intervention group members who improved in this aspect, 65% had a reduction in their A1C at follow-up; only 50–50 improvement in A1C for those whose knowledge did not improve	Community facilities in Hidalgo and Starr counties, Texas
**Rascón 2022 **[45]	In-person Education	Culturally relevant, community-based, *promotores*-led, 6-h intervention to provide guidance on food access, nutrition knowledge, and SNAP	None	Pretest–Posttest Design	Low-income Latino adults	Questionnaire developed by researchers	Preto-follow-up increases in nutrition knowledge (5.05 [SD .14] vs 5.76 [SD.05], *p* < .001. No significant change post vs. follow up) and frequency of consuming fruits (*p* = .007), vegetables (p = .001), and home-prepared meals (*p* < .001)	Health centers and community organizations servicing Latinos across U.S
**Risica 2021 **[46]	In Person Education	Small group sessions delivered by *navegantes* including information about clinical indicators of Type 2 diabetes, hypertension and hypercholesterolemia, and healthy lifestyles	None	Nonrandomized Pretest–Posttest Design	Spanish-speaking, literacy-limited patients	The measures include anthropometries, blood chemistries and an assessment of diet, health and wellness knowledge questionnaire developed by researchers	After the intervention, participants decreased in weight (-1.0 lb), BMI (-0.2 kg/m2), WC (-0.4 in), and cholesterol (-3.5 mg/dl, all *p* < 0.001). Systolic blood pressure decreased (-1.7 mm Hg, *p* < 0.001), and the knowledge score increased (6.8%, *p* < 0.001). No significant changes for A1C or blood glucose levels	Hope Clinic. Providence, Rhode Island
**Romero 2016 **[47]	In-person Education	Culturally tailored 6-week intensive community program targeting CVD health knowledge through weekly, 90-min interactive health sessions	Self-regulation model of disease	Pretest–Posttest Design	Hispanic women, ages 18–85, Spanish speaking and ≥ 1 risk factors for CVD	Heart Disease Knowledge Questionnaire score	Statistically significant (*p* < 0.001) increase in total mean CVD knowledge scores from 39% (mean 11.7/30.0) to 66% (mean 19.8/30.0) postintervention consistent with a 68% increase in overall mean CVD scores. Statistically significant (*p* < 0.001) increase in mean knowledge scores across all five CVD domains	Community health center, Boston, Massachusetts
**Sanchez 2021 **[48]	In Person Education	Six 1-h interactive workshops promoting healthy balanced diets and increased physical activity (PA)	None	Pretest–Posttest Design	Adult Hispanic women responsible for food shopping/preparation	Questionnaire assessing health behavior and outcomes	Increase in nutrition label literacy from baseline to follow-up (percent women who interpreted calorie content per serving size in a container from the nutrition label correctly increased from 51.0% to 77.6% with a similar increase for those who correctly identified daily calorie intake based on serving size. Significant (*p* < 0.001) increase in participants engaging in enough PA to sweat (pre 1.13 [SD 0.84, 1.42] vs post 1.83 [SD 1.52, 2.14]). No significant changes in weight, BMI, or other physical PA levels	Lower Yakima Valley of Washington State
**SotoMas 2015 **[49]	In-person Education	A Multisite Community-Based Health Literacy Intervention for Spanish Speakers	None	Pretest–Posttest Design	Hispanic or Latino, Spanish-speaking adults ≥ 18 years old	TOFHLA	Significant improvement from pretest to posttest in total TOFHLA (73.96 [SD 19.516] vs 83.41 [SD12.057]), raw numeracy (10.86 [SD 4.481] vs 12.94 [SD 3.185]), and reading comprehension (41.51 [SD 7.982] vs 44.71 [SD 4.183]) scores (*p* < 0.0001)	School, church, and hotel. Albuquerque, New Mexico
**SotoMas 2015 **[17]	In Person Education	6-week course combining health literacy content and English-language instruction	Theories of health literacy and health behavior research and practice, sociocultural theories of literacy and communication, and adult learning principles	Randomized Control Trial	Spanish-speaking adults ≥ 21 years old with low-to-intermediate English proficiency	TOFHLA	The intervention group had an average change score on TOFHLA before and after intervention of 12.85 (SD = 10.63, *n* = 77), and in the control group 8.16 (SD = 11.91, *n* = 78), *p* = .01. The mean posttest TOFHLA score was 72.79 (SD = 12.387, *n* = 77) in the intervention group, and 73.69 (SD = 12.437, *n* = 78) in the control group	Community College. El Paso, TX
**Stockwell 2010 **[50]	In Person Education	Three education modules targeted the preceding, enabling and reinforcing factors of care for upper respiratory infections	PRECEDE-PROCEED model	Pretest–Posttest Design	Latino families with young children	Knowledge, Attitudes, Practices (KAP) instrument	Mean composite knowledge/attitude score increased from 4.1 (total: 10) to 6.6 (*p* < .05). Number of parents who reported seeking antibiotics without prescription instead of seeing their health care provider decreased from 6 to 1 (*p* = .06). Families reported other improved care practices (no information on statistical significance)	Columbia University EHS. New York City, NY
**Stockwell 2014 **[51]	In Person Education	Three 1.5-h modules of URI health literacy education for the parent/caregiver who attended Early Head Start (EHS) programs	None	Pretest–Posttest Design	Families attending an infant or toddler EHS group	Pediatric Emergency Department (PED) visits and adverse care practices	Significant less likelihood of visiting the PED when child (age 6 to < 48 months) was ill (8.2% vs 15.7%, *p* = .025), using an inappropriate over-the-counter medication for < 2-year-old child (12.2% vs 32.4%, *p* = .034), or incorrect dosing tool for < 4-year-old child (9.8% vs 31.1%; *p* < .01). No statistically significant difference between groups in having incorrect home remedy beliefs or in use of unprescribed antibiotics for a child < 4 years	4 EHS sites in Manhattan, New York City
**Warren-Findlow 2019 **[52]	In-person Education	Empowering Change in Health Outcomes (ECHO): 2-h evidence-based class regarding education of hypertension, nutrition, and medication use	None	Prospective cohort study	Current hypertension patients ≥ 21 years old who spoke English or Spanish	Hypertension self-care behaviors were measured using a subset of the Hypertension Self-Care Activity Level Effects (H-SCALE)	Significant improvements in diet adherence (*p* < .01) and weight management behaviors (*p* < .05) at 1-moth follow up. Barriers to medication adherence decreased and medication adherence increased (not significant for overall sample). Hispanics had statistically significant improvements in diet (*p* < .01) and weight management behaviors (*p* < .02)	Low-cost health care clinic, Charlotte, NC

**Table 2:** Summary of the study characteristics-multimedia education

**Study**	Intervention type	Intervention description	Theoretical framework	Study design	Target population	Measure of effectiveness	Primary outcomes	Study setting
**Borrayo 2017 **[53]	Multimedia education	8-min narrative film to reinforce desired self-efficacy and behavioral intentions as precursors to engaging in mammography screening	Entertainment-Education framework	Repeated measures design	Latina women	Questionnaires developed by the researchers	Significant increase in BC knowledge (*p* < .001) and mammography self-efficacy (*p* < .01) compared to baseline and control group. Significant time effect in perceived behavioral norms compared to control but no change compared to baseline. No significant changes in behavioral intentions	Community events. Denver, Colorado
**Cabassa 2015 **[54]	Multimedia education	The Secret Feelings *fotonovela*: entertainment-education intervention to break mental health stigmas	Entertainment-Education framework	Randomized Control Trial	Students at three adult night schools in Los Angeles, California	Depression Literacy Questionnaire and test to correctly identify depressive symptoms out of 10 items	Significant increase in depression treatment knowledge scores at posttest (*p* < .001) and 1-month follow-up (*p* < .01). No significant differences in symptom knowledge, social distance, and perceptions of dangerousness	Adult night schools. Los Angeles, California
**Calderón 2014 **[55]	Multimedia education	Animated, culturally sensitive, Spanish video to improve diabetes health literacy (DHL)	None	Randomized Control Trial	Adult Hispanic immigrants with limited diabetes literacy	Short Test of Functional Health Literacy in Adults (S-TOFHLA). Diabetes health literacy was measured by the Diabetes Health Literacy Survey (DHLS)	DHL survey scores improved significantly more in the experimental group than the control group (adjusted mean = 55% vs 53%; *p* = .03). However, DHL survey scores did not differ between experimental and control group participants with marginal or adequate functional health literacy (STOFHLA scores ≥ 17)	South Central Family Health Center. Los Angeles, CA
**Cheney 2023 **[56]	Multimedia education	Tailor MyPlate recipes to local food sources and culture, virtual cooking demonstrations, Spanish cookbook (physcial and electronic), and diabetes education	Analysis, Design, Develop, Implement, and Evaluate (ADDIE) model	Mixed methods	Rural low-income Latinx primary care patients and Indigenous Mexican communities	American Diabetes Association Diabetes Knowledge Questionnaire and adapted Mediterranean Diet Index	Brief exposure to a cooking demo/health education did not increase the total diabetes knowledge score (partly due to high pretest scores). Increased confidence in adherence to two of four components of the Mediterranean diet (baddedsugar = 0.24; 95%CI: 0.02, 0.46; bredmeat = 0.5; 95%CI: 0.02, 0.98)	8 clinics in Eastern Coachella Valley
**Enguidanos 2022 **[57]	Multimedia education	Spanish language patient role model video	Social Learning Theory and the Theory of Reasoned Action	Pretest–Posttest Design	Hospitalized Latino/Hispanic patients diagnosed with chronic life-limiting illness	13-item Palliative Care Knowledge Scale (PaCKS) to assess palliative care knowledge	Significant improvement in palliative care knowledge (pretest mean 6.4, SD = 3.6; posttest mean 11.4, SD = 2.5; *p* < 0.001). Increased intention to enroll family members and themselves in palliative care (64%-98%, *p* < 0.001; and 72%-92%, *p* = 0.01, respectively)	Hospital
**Forster 2016 **[58]	Multimedia education	Bilingual telenovela series on end-stage renal disease (ESRD)	None	Pretest–Posttest Design	Hispanic patients with end-stage renal disease and their families	Knowledge of ESRD test developed by researchers	Significant difference in knowledge scores (control vs treatment) in patients (24.03(4.63) vs 29.94(3.54), *p* < 0.001) and families (22.02(5.76) vs 28.92(4.36), *p* < 0.001) and greater change in behavioral intention scores of patients (6.9(1.72) vs 11.2(1.69), *p* = .02) in the telenovela group	County dialysis clinics and kidney pancreas transplant center
**Gonzalez 2022 **[59]	Multimedia education	Entertainment Education video developed via focus group of target population and 2 professor consultants and focused on behavioral health	Entertainment-Education framework	Randomized Control Trial	Spanish-speaking Latina adult women	Depression Literacy Questionnaire (D-Lit) Depression Stigma Scale (DSS) Attitudes Towards Seeking Professional Psychological Help Short Form (ATSPPH-SF)	Statistically significant higher changes in mean depression literacy scores for intervention vs. control (1.75 ± 0.62 [mean ± standard error], *p* = 0.006). No statistically significant difference in attitudes toward seeking professional psychological help, personal stigma, or perceived stigma	Community center and community health clinic in urban area of a rural state
**Gossey 2011 **[60]	Multimedia education	Audio booklet with visual and narration components with information on how statin medications can reduce risk for cardiovascular events and related topics	Extended Parallel Process Model (EPPM)	Pretest–Posttest Design	Patients with high cholesterol levels, ages 35–75	Survey adapted from previous studies	Significant improvement in posttest (mean difference [SD]) knowledge of statins for African American and Hispanic patients compared with standard of care (2.48 [2.45] vs 0.28 [2.19], *p* < 0.01; and 1.39 [2.78] vs − 0.21 [2.64], *p* = 0.03; respectively). No differences in response efficacy, self-efficacy, personal susceptibility, and severity of the condition	Community health centers in Harris County Hospital District. Houston, TX
**Guiberson 2017 **[61]	Multimedia education	Digital graphic novella that targeted hearing protection devices (HPD) usage, tailored for potentially low literacy and high use of technology and digital media	Pender Health Promotion Model	Pretest–Posttest Design	Spanish-speaking agricultural workers	Survey adapted from previous studies	The posttest scores on Hearing Protection Beliefs statements were significantly better than pretest scores (38 [4.84] vs. 45 [6.12], *p* < 0.01)	Mountain–West region of the U.S
**Gwede 2019 **[62]	Multimedia education	Culturally linguistically targeted Spanish-language *fotonovela* booklet and DVD intervention plus fecal immunochemical test	Preventive health model (PHM)	Randomized Control Trial	Latinos, preferred Spanish, not literate in CRC (aged 50–75)	Screening uptake was evaluated by return of a completed FIT kit Survey adapted from previous studies	The intervention group was associated with greater increases in CRC awareness compared to CDC standard Spanish-language brochure from baseline to follow up scores (5.7[1.9] to 7.9[2.0] vs 5.3[2.4] to 6.4[2.2], *p* = 0.046). and susceptibility (*p* = 0.013). Nonsignificant increase in the average score in the intervention group in contrast to a nonsignificant decrease in the average score in the comparison group regarding behavior and intention of CRC screening. FIT kit uptake did not differ significantly by intervention group	Federally Qualified Health Center. Tampa, FL
**Hernandez 2013 **[63]	Multimedia education	*Fotonovela* presenting adaptive illness perceptions, help-seeking behaviors, depression symptoms, treatment options, and associated misconceptions	Entertainment-Education framework	Pretest–Posttest Design	Spanish-speaking Latina women, ages 18–55,	S-TOFHLA	Statistically significant (*p* < .001) greater increases in pre to post mean depression knowledge scores among the experimental group (Ms = 6.95 and 9.40, respectively) in comparison to the control group (Ms = 7.33 and 7.41, respectively) as well as self-efficacy to identify the need for treatment scores (experimental group: Ms = 8.30 and 12.08, respectively; control group: Ms = 8.80 and 8.98, respectively). No difference in pre to post mean stigma concern about mental health scores. Marginally significant difference in favor of greater intention to seek treatment on intervention group	Large multiservice community clinic; San Francisco, CA
**Merchant 2015 **[64]	Multimedia education	Two arms: HIV/AIDS and HIV testing information delivered orally or through a video in Spanish	None	Randomized Control Trial	Latino adults between18-64 years old	Adapted questionnaire from previous studies	Mean scores on the questionnaire for the video (20.4; 95% CI 19.5 ~ 21.3) and the orally delivered information arms (20.6; 95% CI 19.7 ~ 21.5) were similar (Δ = − 0.15; 95% CI − 1.4 ~ 1.1)	Medical School-affiliated hospital. Providence, Rhode Island
**Molokwu 2017 **[65]	Multimedia education	Culturally sensitive, low literacy, bilingual educational materials delivered by a *promotora*, including CRC screening, and barriers	None	Prospective Controlled Trial	Participants due for CRC screening, ages 50–70 years of age	Validated 10-item knowledge questionnaire that contained questions about CRC screening, risk factors, and warning signs with true/false type responses	Knowledge scores were significantly higher in the intervention group (0.74 vs 0.18, *p* < .0001); so were change in perceived benefits of CRC screening (1.70 vs. 0.35 *p* = 0.0019), perceived susceptibility to CRC (0.4 vs − 0.03, p = .0081), and barriers to screening (4.77 vs − 1.84, *p* < .0001). Significant reduction in the sense of fatalism (− 1.35 vs − 0.66, *p* = .027)	County near the US Mexico border, Texas
**Ochoa 2020 **[16]	Multimedia education	Culturally and linguistically appropriate, Spanish language narrative (storytelling) and nonnarrative film	None	Randomized Control Trial	Spanish-speaking, Mexican-born women, ages 25–45	Questionnaire developed by researchers	The narrative film showed significantly greater knowledge at posttest than its nonnarrative counterpart (5.10 [SD 1.45] vs 4.44 [SD 1.15], *p* = 0.01). However, at follow-up, the main effect of the film was not significant Significantly greater knowledge at posttest (*p* = 0.01). No significant difference at 6-month follow-up (*p* = 0.84). No significant difference in attitudes toward how embarrassing, how physically painful, and how important Pap test were by film condition	Los Angeles County
**Pagán-Ortiz 2021 **[66]	Multimedia education	Website including low literacy text and videos to help manage chronic pain, adapted from an evidence-based workbook	None	Mixed method	Spanish-speaking female adult Latinas with chronic pain	Questionnaire developed by researchers	Statistically significant improvement in knowledge scores postintervention [t(39) = − 10.13, *p* < .001, d = 1.39] and in chronic pain self-efficacy scores from baseline (M = 118.6, SD = 27.9) to follow-up (M = 127.6, SD = 24.6; *p* < .05). No statistically significant decrease in pain severity scores but there was a significant difference in pain interference scores from baseline (M = 4.14, SD = 2.29) to follow-up (M = 3.4, SD = 2.21, *p* < .001)	Community in Greater Boston, Massachusetts area
**Payan 2020 **[67]	Multimedia education	Breast cancer prevention brochure verbally reviewed by a community health worker. Group 1 only received the CUIDARSE brochure, group 2 had a *promotora* deliver the brochure, and group 3 only received the Spanish version of the AHRQ’s guide on reducing the risk of BC	McGuire input–output framework and the health belief model	Randomized Control Trial	Women ≥ 35 years	Questionnaire developed by researchers	Significant (p ≤ .05) changes in BC knowledge in all groups (8 points at baseline, increases (+ 3) in mean knowledge scores at postintervention, and decrease (-1) point in score at 3 months). Knowledge of perceived BC susceptibility increased postintervention and at 3 months for Group 1 (54.6% to 65.1%), Group 2 (67.1% to 70.7%), and the control (53.3% to 62.5%). These changes were significant (*p* ≤ .05) except for Groups 1 or 2 at 3 months. Treatment effects were not significant. Self-efficacy increased significantly for Group 1 (72.2% to 87.3%), Group 2 (69.2% to 88.6%), and the control (64.6% to 80.3%). At 3 months, rate decreased for Group 1 and did not change for Group 2 and control group	Public Hospital. California
**Phipps 2018 **[68]	Multimedia education	10-week culturally applicable science and health curriculum delivered through Spanish-language radio	None	Pretest–Posttest Design	Spanish-speaking adults ≥ 18 years old	SAHLSA-50, Rapid Estimate of Adult Literacy in Medicine—Short Form (REALM-SF), and NVS assessments	Significant increases in health and science knowledge (mean score 68.4% preintervention, 95% CI: 63.7 to 73.0 versus mean score 77.0% posttest, 95% CI: 73.6 to 80.5)	Rhode Island
**Ramos 2013 **[69]	Multimedia education	General health education curriculum in the areas of cardiovascular disease (CVD), nutrition (N), diabetes (D), metabolic syndrome (MS), and sexually transmitted diseases (STD)	Train-the-trainer model of community education	Pretest–Posttest Design	Underserved Hispanic males	Questionnaire developed by researchers	Improvements in all areas in pretest vs. posttest (CVD pretest 16/26 participants scoring > 40% vs posttest 20/38 participants scoring > 80%; N pretest 20/25 scoring below 50% vs posttest 20/25 participants scoring > 80%; D pretest most of 26 participants scoring 20–40% vs posttest 24/26 scoring > 80%; MS pretest 9/18 scoring at 20% vs posttest 6/18 scoring > 80%; STD pretest 17/22 scoring < 30% vs posttest 20/22 scoring > 80%.)	Faith-based organizations in Shelbyville, KY
**Reuland 2012 **[70]	Multimedia education	Multimedia decision aid including a 14-min Spanish-language video, a printed brochure with an overview and rationale for CRC screening, and specific information about colonoscopy and FOBT	Prochaska’s Transtheoretical Model and Social Cognitive Theory	Pretest–Posttest Design	Hispanics, ages 50–75, who are not fluent in English	Questionnaire adapted from previous studies	Participants' knowledge scores increased from 20 to 72% after decision aid viewing ([95%CI]: 52% [70, 71]). The proportion with high screening self-efficacy increased from 67 to 92% (25% [13, 57]); the proportion with high screening intent increased from 63 to 95% (32% [21, 63]). All differences were statistically significant at *p* < 0.001	FQHC in Caswell County, NC, and an academic medical center in central NC
**Riera 2017 **[72]	Multimedia education	Spanish asthma educational video about asthma types, medication recognition and delivery, home management steps, caregiver consistency, and how to deal with cigarette smoke exposure	None	Cross-Sectional Design	LEP Latino asthma caregivers	Validated Spanish asthma knowledge questionnaire	Mean baseline asthma knowledge scores improved from 58.4 to 66.4 (95% CI 5.3–10.7; *p* < 0.01)	Academic children’s hospital and local community center. Fair Haven, Connecticut
**Robinson 2015 **[73]	Multimedia education	Culturally sensitive, electronic, interactive sun-protection educational video program. Participants used headphones to listen to the program in the waiting room and were Non-Hispanic white, Hispanic/Latino, or Non-Hispanic black	Transtheoretical model and Tansportation theory	Randomized Control Trial	Hispanic adults who received a kidney transplantation within the past 2–24 months	S-TOFHLA Self-report survey on Sun-Protection Behavior	Hispanic participants had the greatest increase in knowledge from pretest (2 [SD 0.2]) to posttest (8 [SD1.1]), *p* < .05. The rest of the participants also saw a significant increase. Non-Hispanic black participants had no significant reduction in outdoor exposure. Overall, all participants improved attitudes and sun protection at 2 weeks but these results varied between groups	Northwestern Medicine and University of Illinois health systems. Chicago, IL
**Sanchez 2019 **[74]	Multimedia education	*Fotonovela* using an entertainment-education approach, and discussion with a bilingual LCSW serving as the Depression Educator	None	Pretest–Posttest Design	Hispanic adults	9-item Patient Health Questionnaire (PHQ-9), Depression Knowledge Measure (DKM) and three stigma measures	Stigma Concerns About Mental Health Care (F [1.907, 574.076] = 21.914, *p* < .001, partial ƞ2 = .068) significantly decreased over time. Social distance scores significantly increased (F [1.823, 548.637] = 50.288, *p* < .001, partial ƞ2 = .143), as did depression knowledge (F [1.886, 563.768] = 807.305, *p* < .001, partial ƞ2 = .730) and antidepressant stigma scores (F [2, 588] = 14.633, *p* < .001, partial ƞ2 = .047)	Federally Qualified Health Center, Texas
**Schlumbrecht 2016 **[26]	Multimedia education	10-min PowerPoint video about ovarian cancer delivered to participants enrolled in in family literacy programs	None	Cross-Sectional Design	Hispanic women enrolled in family literacy programs	Questionnaire developed by researchers	In the pretest group, 45% of questions were answered correctly versus 84% in the posttest group (*p* < 0.001)	Mesa Public Schools Family Literacy Program. Mesa, Arizona
**Unger 2013 **[25]	Multimedia education	*Fotonovela* about depression symptoms, treatment, and stigma surrounding it among Hispanics	None	Randomized Control Trial	Adults attending a community school	Questionnaire developed by researchers and D-Lit	At posttest, *fotonovela* group had significantly higher effects in depression knowledge scores (.23 *p* < . 05) and significantly lower antidepressant stigma scores (-.33 *p* < . 05) compared with the students who read the text pamphlet. By the 1-month follow-up, these effects had become nonsignificant, but the *fotonovela* group had significantly lower mental health care stigma (-.15 *p* < . 05) than the text pamphlet group. For the other variables, there was no significant difference between the two groups at posttest or 1-month follow-up (willingness to seek help and self-efficacy)	Los Angeles Unified School District. Los Angeles, California
**Valdez 2018 **[75]	Multimedia education	Education delivered through an interactive, multimedia kiosk about cervical cancer risk factors as well as attitudes and self-efficacy regarding screening	Transtheoretical model of health behavior change	Randomized Control Trial	Latinas, ages 21–69 years,	Questionnaire adapted from previous studies	Increased knowledge (*p* < 0.0001) and more favorable attitudes at follow-up; fewer intervention group women never thought of getting a pap test (46 vs. 54%, *p* = 0.050) or agreed that it is fate whether a woman gets cervical cancer or not (24 vs. 31%, *p* = 0.043). The groups did not differ significantly on the proportion who had obtained or made an appointment for a pap test at follow-up (51 vs. 48%, *p* = 0.35) or in reported levels of self-efficacy regarding pap screening (> 90% in both groups at pre and posttest) at postintervention	Community clinics. Los Angeles, San Jose and Fresno, CA
**Valenzuela-Araujo 2021 **[76]	Multimedia education	Culturally and linguistically tailored 9-min educational video about healthcare navigation and engagement	None	Cross-Sectional Design	Spanish speaking guardians of infants < 2 months	NVS	Significant increase in knowledge scores after viewing the educational video (mean 2.9 pretest vs 4.3 posttest *p* < 0.001), with knowing appropriate fever criteria based on child age having the greatest improvement (46.8–96.2% correct)	General pediatrics clinic. Baltimore, MD
**West 2014 **[77]	Multimedia education	4-min educational video in Spanish explaining the procedures patients undergo during their anesthetic care	Iowa Model of Evidence-Based Practice to Promote Quality Care	Pretest–Posttest Design	Adult patients with ASA physical status 1, 2, and 3, scheduled for elective surgery	Assessment of anxiety, knowledge, and satisfaction was obtained using a visual analog scale (VAS)	Significant reduction in anxiety score in intervention group (median reduction 2 vs 0; *p* = 0.020). Increase in satisfaction score in the video group (median increase 2 vs 0; *p* = 0.046). No difference in reported knowledge-improvement scores between the two groups (3.5 vs 4; *p* = 0.908). In Spanish-speaking patients, the addition of an instructional video in Spanish to a preanesthesia interview decreased anxiety and increased patient satisfaction	Massachusetts General Hospital

**Table 3:** Summary of the study characteristics multimodal

**Study**	Intervention type	Intervention description	Theoretical framework	Study design	Target population	Measure of effectiveness	Primary outcomes	Study setting
**Calderon 2022 **[78]	Other	Workshop including a short video on possible psychotic and depressive symptoms, La CLAve mnemonic device to describes the main symptoms of psychosis, and a narrative film to discuss its portrayal of symptoms	Mental health knowledge, self-efficacy, illness attributions, and help-seeking model	Pretest–Posttest Design	Latinx/endorse being born in Latin-American countries	Questionnaires adapted from previous studies which included some rating scales and mostly open-ended questions to minimize priming	Significant increase in psychotic symptoms reported as definition of serious mental illness (pre, M = 0.69, SD = 0.61; post, M = 1.23, SD = 0.90; *p* < 0.001) and ability to detect a serious mental illness in others (*p* < 0.05), in ability to detect a serious mental illness in others (pretraining: M = 2.83, SD = 1.31; posttraining: M = 3.24, SD = 1.27, *p* < .05), and decrease in participants' recommendations for nonprofessional help-seeking (pre: 49.4%, post: 25.9%, p = .001). No significant change in recommendations for professional help (pre: 64.2%, post: 72.8%, *p* = .25)	Local churches, community centers, and schools in Los Angeles, California
**Chalela 2018 **[79]	Other	Choices included three components: an educational interactive video, a low-literacy booklet, and care coordination by patient navigation	Social Cognitive Theory and the Stages of Change Model	Randomized Control Trial	Latina breast cancer patients ≥ 18 years old	Survey developed by researchers	Significant change on agreement with stages of readiness statements (*p* < .002)	UT Health San Antonio Cancer Center. San Antonio, TX
**Cullen 2022 **[80]	Other	Resident-led multidisciplinary quality improvement team developed intervention that included pictogram medication dosing instructions, teach-back, aid and educational materials	Model for Improvement and the PDSA (Plan, Do, Study, Act) cycles	Prospective cohort study	Caregivers and patients	Nursing teach-back assessments developed by researchers	Strong correlation between provision syringes and accurate medication administration (R = 0.84). Overall understanding of liquid acetaminophen administration instructions (report of medication name, purpose, dose, and frequency) improved from 39.8% to 74%	Publicly insured primary care clinic affiliated with a large tertiary medical center
**Dunlap 2015 **[81]	Other	Clinical encounters in which the patient interacts with someone who speaks the same native language at all times while in the clinical setting	None	Prospective cohort study	Families of Hispanic pediatric patients	Survey developed by researchers	Spanish speaking patients receiving care in Spanish showed the highest level of satisfaction (M = 6.91, S.D. = 0.30 *p* < 0.01) compared to using interpreters or to English speaking patients, they also had the highest perceived quality of information transfer during the visit (M = 6.91, S.D. = 0.20, *p* < 0.001)	Lucile Packard Children’s Hospital Stanford, California
**Lajonchere 2017 **[71]	Other	Science Briefs to improve parental understanding of evidence-based causes and emerging treatments for Autism Spectrum Disorder in published biomedical research	None	Randomized mixed method	Hispanic parents or their spouse/partner with ≥ 1 child with Autism Spectrum Disorder	Questionnaire developed by researchers	Increase prepost proportion of correct answers in knowledge test (0.46 [SD = 0.15] to 0.65 [SD = 0.16], *p* < 0.001)	Community based organization, region not specified
**Mohan 2015 **[82]	Other	PictureRx illustrated medication list depicting the medication, indication, and dosing instructions, accompanied by plain language bilingual text	None	Randomized Control Trial	Latinos with diabetes	Medication Understanding Questionnaire (MUQ)	Statistically significant difference in medication understanding between the PictureRx group (86.4 [SD, 12.6]) and the usual care group (76.4 [SD, 18.0]), the adjusted difference was 9.9 (95% CI, 5.7–14.2; *p* < .001). Not statistically significant increase in medication adherence in the intervention group (0.5 [95% CI, —0.1 to 1.1])	Safety net clinic. Nashville, TN
**Vadaparampil 2022 **[83]	Other	In-person workshop and online curriculum to facilitate identification, referral, and navigation of Latinas to genetic counseling/testing	Adult Learning Theory	Pretest–Posttest Design	Bilingual community outreach and education professionals providing services to Latinas	Validated HL tools	Significant increase in hereditary breast and ovarian cancer knowledge (*p* = 0.002), genetic health literacy for the Familiarity component (*p* < .0001), and self-efficacy (*p* < .0001)	Online portion and unspecified in-person location

Topics included prenatal care and parent education; breast, cervical, colorectal, and ovarian cancer; diet and healthy lifestyle choices; mental health literacy; diabetes; cardiovascular disease; end-stage renal disease; asthma; upper respiratory infections; inflammatory bowel disease; HIV/AIDS; skin care; hearing loss prevention; medication understanding; palliative care; family health history; chronic pain; healthcare navigation; and anesthesia education. Thirty-four studies employed a theoretical framework when designing and conducting research, and there was little heterogeneity in terms of frameworks employed. No framework was shared by more than four studies.

Studies were performed in a variety of settings, including clinics (*n* = 13), hospitals and health centers (*n* = 13), Federally Qualified Health Centers (FQHCs) or safety net clinics (*n* = 9) and community spaces (*n* = 18). Common community settings, which include community health centers and safety net clinics, frequently used curricular interventions embedded in educational curricula and educational workshops (*n* = 18). Larger hospital networks implemented organizational interventions, often updating their practices or replacing standard-of-care materials with language and culturally concordant materials (*n* = 8).

### Measures of effectiveness

The measures of successful enhancement of HL used by the studies in our review were heterogenous, and were often unvalidated measures of knowledge or beliefs. Twenty-two studies had a questionnaire about beliefs, knowledge or practice that was developed by the researchers, limiting the validity of their results. Fifty-eight studies measured effectiveness quantitatively, and four were mixed methods. The two most common approaches to primary outcomes were either HL assessment tools [16, 17, 25, 25, 26, 28, 30–32, 34–38, 41, 47, 49, 50, 53–61, 63–72, 74–76, 78–83] (*n* = 45) or health outcomes [27, 29, 39, 42, 48, 51, 52] (*n* = 7), with some studies using both [15, 43, 46, 84–90] (*n* = 10). HL tools most commonly took the form of pretest/posttest questionnaires specifically developed by the researchers to assess knowledge gained over the course of a given intervention. A few studies (*n* = 10) utilized previously validated disease-specific assessments of HL, such as the High Blood Pressure-Health Literacy Scale for high blood pressure [32], or more standardized Test of Functional Health Literacy in Adults (TOFHLA) [31, 37, 38, 49, 50, 52, 55, 60, 68, 73] (*n* = 10) and/or Newest Vital Sign (NVS) [28, 43, 51, 52, 68, 76] (*n* = 6), to assess overall changes based on the participant’s ability to read and understand generic health-related materials.

Other outcome measures included patient satisfaction and patient attitude surveys, which were intended to predict not only knowledge of health conditions but also attitudes toward receiving treatment [84]. Higher satisfaction and improved attitude scores were thought to lead to a more positive and confident approach in obtaining healthcare. Some studies measured improvements in confidence and self-advocacy [31, 34, 70]. Medical health measurements and outcomes, such as blood pressure readings, were also commonly used as primary outcomes [27, 46, 48, 74]. Secondary measures were also varied and included measures of patient confidence, perceived support, perceived barriers to care, level of comfort, and adherence to the intervention.

Studies also varied in how they measured the long-term changes associated with their interventions. Thirty-one studies had a follow up of at least a month, ranging from 1 to 24 months, with most studies doing a 1 month follow up (*n* = 7) or a 3 month follow up (*n* = 8).

#### Overview of health literacy interventions

##### In-Person Education

In-person education health literacy programs varied in presentation of material but shared commonalities of repeated meetings in a class setting that encouraged practice and facilitated opportunities for enhanced participant engagement compared to other modalities (Table 1). A study by Cruz [30], found the use of 90 min training session conducted by *promotores* focusing on general knowledge for diabetes, risk factors, and prevention and control of diabetes provided significant improvement on diabetes knowledge for diabetic participants comparing pre- and posttest scores (13.7 vs. 18.6, *p* < 0.001; Cohen’s d = 1.2), and for nondiabetic participants (12.9 vs. 18.2, *p* < 0.001; Cohen’s d = 1.2).

Similarly, Buckley [27] assessed the implementation of social clubs hosted by *navegantes* (patient navigators) for 2 h every week over 5 weeks. The findings suggested 88.9% of 126 participants increased health literacy and over 60% decreased at least one risk factor associated with metabolic syndrome. Change for those that improved, [mean (SD)]: Weight [− 6.0 lbs (5.2)]; BMI [− 1.1 (1.0)]; Waist Circumference [− 2.2 inches (1.5)]; Blood Glucose [− 26.3 mg/dl (27.5)]; LDL Cholesterol [− 19.1 mg/dl (16.8)]; Systolic BP [− 11.1 mmHg (9.5)]; Health Literacy Test (*n* = 117) [+ 22.2% (19.7%)]. Castaneda [28] studied the implementation of 6-week, culturally tailored, *promotora*-based group for health prevention knowledge and found participants improved their self-reported cancer screening, breast cancer knowledge (Mpre = 2.64, Mpost = 3.02), daily fruit and vegetable intake, and ability to read a nutrition label (*p* < 0.05).

Across all the different in-person education there were common findings that repeated exposure to health education information in an engaging classroom setting provided meaningful improvements to health literacy in SLP populations that correlated with improvements in physical health and greater utilization of health screening services.

##### Multimedia education

Multimedia approaches to health literacy education varied from narrative films and *fotonovelas* to animated culturally sensitive videos and virtual workshops to assess applied knowledge (Table 2). The commonality shared with these interventions were that they could largely be independently navigated without need for transportation or cost to the participant as long as they had access to a computer and the internet.

A study related to health literacy in women’s health, Borrayo [53] found that through a 8-min narrative film to reinforce desired self-efficacy and behavioral intentions as precursors to engaging in mammography screening there was a significant increase in breast cancer knowledge ( Wilks’s Λ = 0.75, F(1, 39) = 13.15, *p* < 0.001, η2 = 0.25) and mammography self-efficacy ( Wilks’s Λ = 0.76, F(1, 37) = 11.64, *p* < 0.01, η2 = 0.24) compared to baseline and control group. Furthermore, Cabassa [54] assessed the use of a *fotonovela* centered around entertainment-education intervention toward mental health stigma finding a significant increase in depression treatment knowledge scores at posttest ( B = 1.22, *p* < 0.001, Cohen’s d = 0.91) and 1-month follow-up ( B = 0.81, *p* < 0.01, Cohen’s d = 0.53). Calderon [55] looked at the implementation of an animated, culturally sensitive, Spanish video to improve diabetes health literacy (DHL). The findings reported DHL survey scores improved significantly more in the experimental group than the control group (adjusted mean = 55% vs 53%, F = 4.7, df = 1, *p* = 0.03). Additionally, Cheney [56] studied the application of tailor MyPlate recipes to local food sources and culture, virtual cooking demonstrations, and Spanish cookbook, on diabetes education finding there was an increased confidence in adherence to two of four components of the Mediterranean diet (b_added sugar_ = 0.24; 95%CI: 0.02, 0.46; b_redmeat_ = 0.5; 95% CI: 0.02, 0.98).

##### Other types of multimodal strategies

 Multimodal strategies provided a crossover between in-person and multi-media focused health literacy approaches (Table 3). A study by Auger [15], found the use of *fotonovelas* as an educational tool along with health education facilitation by the teacher and lay health educator provided an increased knowledge of pregnancy, childbirth, and breastfeeding (*p* < 0.001) and confidence in navigating pregnancy, caring for oneself and the baby, and interacting with health professionals (*p* ≤ 0.05).

Additionally, Calderon [78] took a multimodal approach to mental health education via workshops including a short video on possible psychotic and depressive symptoms, La CLAve mnemonic device to describe the main symptoms of psychosis, and a narrative film to discuss its portrayal of symptoms. That study demonstrated a significant increase in psychotic symptoms reported as definition of serious mental illness (pre, M = 0.69, SD = 0.61; post, M = 1.23, SD = 0.90, t(80) = − 5.64; *p* < 0.001; Cohen's d = 0.70) and ability to detect a serious mental illness in others (pretraining: M = 2.83, SD = 1.31; posttraining: M = 3.24, SD = 1.27, t(74) = − 2.76, *p* < 0.05; Cohen's d = 0.32), and decrease in participants' recommendations for nonprofessional help-seeking (pre: 49.4%, post: 25.9%, *N* = 81, *p* = 0.001). There was no significant change in recommendations for professional help (pre: 64.2%, post: 72.8%, *N* = 81, *p* = 0.25).

### Reported effectiveness by intervention type

Of the interventions, 89% of in-person educational interventions (*n* = 25) and 89% of multimedia educational interventions (*n* = 24) found improvements to HL. All multimodal interventions (*n* = 7) provided improvements in HL. The use of lay health advisors and *promotores* was correlated with increased effectiveness; all 18 studies that used this technique reported that their interventions had caused statistically significant changes in HL [27, 28, 34, 46, 70]. Similarly, all nine of the studies implementing *fotonovela* strategies reported statistically significant improvements in HL [16, 25, 53, 54, 61, 63, 74].

### Quality assessment

The risk of bias assessment for RCTs evaluated risks due to randomization, outcomes, and result reporting (Table 4). Among RCTs (*n* = 17), one was assessed as having a high risk of bias, and eight were assessed as having some concerns. Non-RCTs were likewise evaluated for risk of bias due to problems with recruitment, confounding factors, missing data, and selective measurement of outcome or result reporting (Table 5). Among non-RCTs (*n* = 36), 14 studies had a serious risk of bias, while the remaining 22 studies had a moderate risk of bias.

**Table 4:** Risk of bias summary for randomized studies

**Authors (year of publication)**	**Bias in the randomization process**	**Bias due to deviations from the intended interventions (effect of assignment to intervention)**	**Bias due to deviations from the intended interventions (effect of adhering to intervention)**	**Bias due to missing outcome data**	**Bias in measurement of the outcome**	**Bias in selection of the reported result**	**Overall Bias**
Mas et al., 2018 [38]	Some Concerns	Low	Low	Low	Low	Low	Some Concerns
Jandorf et al., 2012 [33]	Low	Low	Some Concerns	Low	Low	Some Concerns	Some Concerns
Stockwell et al., 2014 [51]	Low	Low	Low	Low	Low	Low	Low
Hernandez et al., 2013 [63]	Low	Low	Low	Low	Low	Low	Low
Borrayo et al., 2017 [53]	Low	Low	Low	Low	Low	Low	Low
Auger et al., 2015 [15]	Low	Low	Low	Low	Low	Low	Low
Gossey et al., 2011 [60]	Low	Low	Low	Low	Low	Low	Low
West et al., 2014 [77]	Low	Some Concerns	Low	Low	Low	Low	Some Concerns
Ockene et al., 2012 [42]	Low	Some Concerns	Low	Low	Low	Low	Some Concerns
Calderón et al., 2014 [55]	Low	Low	Low	Low	Low	Low	Low
Chalela et al., 2018 [79]	Low	Low	Low	Low	Low	Low	Low
Chen et al., 2022 [29]	Low	Low	Low	Low	Low	Low	Low
Howie-Esquivel et al., 2014 [31]	Some Concerns	Low	Low	Low	Some Concerns	Low	Some Concerns
Gonzalez et al., 2022 [59]	Some Concerns	Low	Low	Low	Low	Low	Some Concerns
Gwede et al., 2019 [62]	Low	Low	Low	Low	Low	Low	Low
Merchant et al., 2015 [64]	Low	Low	Low	Low	Low	Low	Low
Arun et al., 2022 [82]	Low	Low	Low	Low	Low	Low	Low
Ochoa et al., 2020 [16]	Low	High	Low	Low	Low	Low	Some Concerns
Otilingam et al., 2015 [43]	Low	Some Concerns	Some Concerns	Low	Low	Low	Low
Payán et al., 2020 [67]	Low	Low	Low	Low	Low	Low	Low
Robinson et al., 2015 [73]	Low	Low	Low	Low	Low	Low	Low
Mas et al., 2015 [17]	Some Concerns	Some Concern	Some Concerns	Low	Low	Low	Some Concerns
Unger et al., 2013 [25]	Low	Low	Low	Low	Low	Low	Low
Valdez et al., 2018 [75]	Low	Low	Low	Low	Low	Low	Low
Cabassa et al., 2015 [54]	Low	Low	Low	Low	Low	Low	Low
Molokwu et al., 2017 [65]	High	Low	Low	High	Some Concerns	Low	High

**Table 5:** Risk of bias summary for nonrandomized studies

**Authors (year of publication)**	**Bias due to confounding**	**Bias in selection of participants into the study**	**Bias in classification of interventions**	**Bias due to deviations from intended interventions**	**Bias due to missing data**	**Bias in measurement of outcomes**	**Bias in selection of the reported result**	**Overall risk of bias**
Cheney et al., 2023 [56]	Moderate	Low	Low	Low	Low	Moderate	Low	Moderate
Han et al., 2018 [32]	Serious	Low	Low	Low	Moderate	Low	Low	Serious
Soto Mas et al,. 2015 [49]	No information	Low	Low	Low	Low	Low	Low	Moderate
Guiberson et al., 2017 [61]	Moderate	Low	Low	Low	Low	Low	Low	Moderate
Castañeda et al., 2016 [28]	Moderate	Low	Low	Low	Low	Low	Low	Moderate
Riera et al., 2017 [72]	Moderate	Low	Low	Low	Low	Low	Low	Moderate
Soto Mas et al., 2017 [37]	Moderate	Low	Low	Low	Low	Low	Low	Moderate
Peña-Purcell et al., 2014 [44]	Moderate	Low	Low	Serious	Low	Low	Low	Serious
Mojica et al., 2016 [39]	Moderate	Low	Low	Low	Low	Low	Low	Moderate
Risica et al., 2021 [46]	Moderate	Low	Low	Moderate	Low	Low	Low	Moderate
Rascón et al., 2022 [45]	Moderate	Low	Low	Low	Low	Low	Low	Moderate
Sanchez et al., 2019 [74]	Moderate	Low	Low	Low	Low	Low	Low	Moderate
Ramos et al., 2013 [69]	Serious	Low	Low	Low	Low	Low	Low	Serious
Vadaparampil et al., 2022 [83]	Moderate	Low	Low	Moderate	Moderate	Low	Low	Moderate
Sanchez et al., 2021 [48]	Moderate	Low	Low	Low	Low	Serious	Low	Serious
Warren-Findlow et al., 2019 [52]	Moderate	Low	Low	Low	Serious	Serious	Low	Serious
Kaphingst et al., 2011 [34]	Moderate	Low	Low	Low	Low	Low	Low	Moderate
Calderon et al., 2022 [78]	Moderate	Low	Low	Low	Low	Low	Low	Moderate
Forster et al., 2016 [58]	Moderate	Low	Low	Low	Low	Low	Low	Moderate
Pagán-Ortiz et al., 2021 [66]	Moderate	Low	Low	Low	Low	Low	Low	Moderate
Stockwell et al., 2010 [50]	Serious	Low	No Information	Low	Low	Moderate	Low	Serious
Cullen et al., 2022 [80]	Serious	Low	Low	Moderate	Moderate	Low	Low	Serious
Moralez et al., 2012 [40]	Moderate	Low	Low	Low	Low	Moderate	Low	Moderate
Enguidanos et al., 2022 [57]	Moderate	Low	Low	Low	Low	Low	Low	Moderate
Valenzuela-Araujo et al., 2021 [76]	Moderate	Low	Low	Low	Low	Low	Low	Moderate
Laughman et al., 2017 [35]	Serious	Low	Low	Low	Low	Low	Low	Serious
Phipps et al., 2018 [68]	Moderate	Low	Low	Low	Low	Low	Low	Moderate
Schlumbrecht et al., 2016 [26]	Serious	Low	Low	Moderate	Moderate	Low	Low	Serious
Reuland et al., 2012 [70]	Moderate	Low	Low	Low	Low	Low	Low	Moderate
Cruz et al., 2013 [30]	Moderate	Low	Low	Low	Low	Low	Low	Moderate
Dunlap et al., 2015 [81]	Moderate	Low	Low	Low	Low	Low	Low	Moderate
Martin et al., 2016 [36]	Serious	Low	Low	Low	Low	Low	Low	Serious
Nitsos et al., 2017 [41]	Serious	Low	Low	Moderate	Low	Moderate	Low	Serious
Buckley et al., 2015 [27]	Serious	Low	Low	Serious	No information	Low	Low	Serious
Romero et al., 2016 [47]	Serious	Low	Low	Serious	Moderate	Low	Low	Serious
Lajonchere et al., 2016 [71]	Serious	Low	Low	Low	Low	Low	Low	Serious

Supplementary Table 1

## Discussion

To the best of our knowledge, this review is the first to systematically describe and evaluate the effectiveness of HL interventions among patients with SLP in the United States. Recent reviews have studied the impact of different intervention strategies for increasing the HL of the general population [85] and for immigrant communities [18] but have not focused on Spanish speakers – a community largely at risk for low HL and poor health outcomes [5, 11, 17].

Our review found that, as with other populations with a non-English language preference, including migrant populations [86], there is a lack of evidence-based specific interventions to raise HL tailored to U.S. patients with SLP. Further, our review found that the few existing studies may be at risk of bias. The high risk of bias we found especially in non-RCTs on this topic likely represents both the lack of attention to research addressing this need in SLP populations, as well as difficulties inherent in testing and measuring interventions aimed at improving HL more broadly. Our review of quality was in line with other reviews on this topic [18, 87] which found that a risk of bias was introduced primarily due to difficulty blinding participants and moderators due to the nature of study designs. This made RCTs more difficult to conduct, and as a result, studies primarily used pretest/posttest and cross-sectional methodologies. This finding points not only to a need for high-quality studies of HL in this population, but also for the potential to critically rethink how to conduct research on HL in a high-quality, low risk of bias way. Additionally, studies reported sample sizes ranging from 10 to 943 participants, which made it difficult to compare effect sizes directly. This variation likely reflects the dissimilarity of study designs, sample populations and setting types, thus making it difficult to compare across studies, a challenge that has been previously acknowledged for reviews of HL.

There are also significant differences in patient populations across reviews, and many studies had a low number of participants. This small sample size was in some cases due to strict study inclusion criteria, and other cases were due to high rates of attrition. This could be partly due to primarily targeting participants already facing cultural, socioeconomic, and educational barriers, making them more difficult to recruit and retain in research. Many studies have indicated that their sample may not be representative due to sampling methodologies or that there may be limited generalizability of results. This was due in part to convenience sampling or small sample sizes, which made it difficult to determine whether findings represented a true effect due to limitations in statistical power.

Some studies were focused on only one research site and/or a highly specific Hispanic immigrant community with a SLP (i.e., Mexican immigrants [52]), limiting generalizability. At the same time, while we attempted to capture the difference between the broader category of Spanish speaking populations in the United States and specific migrant populations, most studies did not include this sort of information, indicating a potential need for studies that focus on specific SLP migrant communities. No studies addressed pediatric populations. Another factor limiting the generalizability of the reviewed studies was that the majority of study participants were women; this may be tied to a wider lack of healthcare utilization among Hispanic men, including those with a SLP [88–90]. The relative paucity of males in the sample population of the studies may indicate a need for research that focuses on men with a SLP. To date, only a few strategies have been developed to include males with a SLP in research, including the use of male community health workers and health outreach in workplaces and providing public transportation [41]. Finally, the studies reviewed included a predominantly adult to middle-aged population (aged 30–50) rather than older adults who are more at risk for serious medical problems. This suggests that several important populations (men, children, older adults) may be missed by most previous HL interventions in populations with SLP.

The varied, poorly standardized, heterogenous measures used to assess HL in reviewed studies demonstrate that HL as a concept is poorly defined by researchers, and the concept likely encompasses more than can be quantified by numeric scores on standardized assessments of knowledge. For example, in assessing HL, there may also be a need to address the ability of patients to advocate for themselves, ask questions, and feel empowered to change their health behaviors [8, 9, 16, 74, 85]. Existing measures of HL may not fully capture HL concepts, and thus may be a poor proxy of effectiveness. Studies in our review often used measures that were not validated and tested knowledge on a specific health topic or reported beliefs about health as proxies for HL, and relatively few measured direct behavioral changes, attempts at communication self-efficacy or advocacy, or effects on health outcomes. A key takeaway of this review is the need to critically reexamine definitions and measures of HL, and to develop and validate improved qualitative and quantitative measures of HL outcomes. Only about half of the studies used a theoretical framework to inform their intervention or research, and studies rarely employed the same frameworks, perhaps partially accounting for the variety of measures and the limitations in the ways that HL was framed by researchers.

We found that studies of HL among people with SLP in the United States therefore followed trends within the literature, in which HL is measured through knowledge of health/healthcare systems; to a lesser extent, studies included in our review also attempted to measure participants’ use of information related to health and healthcare, and their ability to maintain health through collaboration with health providers [7]. When framed in terms of Nutbeam’s health outcomes approach, the studies mostly attempted to measure functional HL, occasionally addressed communicative/interactive HL, and rarely attempted to address critical HL [8, 9]. This focus on HL as knowledge rather than personal health advocacy has important ramifications in terms of the skills that HL interventions focus on building, and may help to explain the success and failure of HL interventions.

In addition to the importance of improving individual’s health literacy there is support in the literature to improve “organizational health literacy.” Organizational health literacy refers to the responsibility for health care systems to address populations with low health literacy [91]. Methods for organizations to address populations with low health literacy include “reducing the complexity of health care; increasing patient understanding of health information and enhancing supports for patients of all levels of health literacy” [91]. Because limited health literacy has been associated with increased cost of healthcare organizations have an incentive to address health literacy. However, few if any of the studies attempted to address organizational health literacy, and placed the onus for building HL on the individual patient and their family.

### Specific recommendations

Successful interventions focused on HL interventions that targeted SLP populations through linguistically and culturally concordant techniques that utilized community member liaisons and culturally relevant storytelling. Successful interventions were also often well integrated within communities and organizations.

Our review found that interventions utilizing cultural and linguistic concordance (ie. Spanish-language, culturally salient concepts/terms), liaison roles (*promotores*), and narrative media were effective in achieving notable improvements in HL among patients with SLP. These interventions focused more on what Nutbeam frames as communicative/interactive HL [8, 9]. The relative success of these interventions may be due to more effective communication with patients through a shared cultural background and deeper levels of trust. One particularly effective strategy is the use of narrative in media, as seen with *fotonovela* strategies [16, 25, 53, 54, 61, 63, 74]. Such strategies may involve a video or booklet presenting important health information in a story format. Narrative media appeared to activate study participants and lead to improvements in health knowledge and behavior change. Another important element of effective multimedia health interventions is cultural adaptation to address previously identified cultural concepts such as *respeto*, *familismo*, *marianismo*, and *personalismo* [16, 25, 55, 62, 65, 68, 73, 76]. Realistic stories with Spanish-speaking characters and culturally tailored information were key components of these interventions [25, 54, 55, 74]. Prior research has shown that identification with storytellers is an important prerequisite for patient engagement and is particularly useful in combating cultural stigma and eliciting behavioral health changes [92].

Liaison roles that employ educators and health promoters from similar cultural backgrounds as patients were also an important strategy used by reviewed studies. The lived experience and cultural understanding from these workers (*promotores*, *navegantes*, community health workers) may help boost patient comprehension and overcome distrust of the healthcare system [93]. Linguistic and culturally concordant care, including cultural competency training for providers, has also repeatedly been identified as a successful strategy for increasing HL among immigrant populations generally [18]. Furthermore, successful interventions often consider the opinions of the target population when designing content to ensure that the experience is culturally relevant [16, 28, 29, 34, 35, 40, 44, 45, 55, 62, 65, 73, 76].

Our review also included a number of multimedia intervention strategies (*n* = 22) that might be utilized more often in the future following the increased acceptance of online options since the COVID-19 pandemic. Interestingly, of the 14 studies published since 2020, seven were multimedia interventions. Our search also revealed the importance of including nonmedical settings such as community gathering spaces, which may serve as a hub for creating a wider network of health promotion. The integration of health promotion interventions into communities may be complimentary to the long-term reinforcement of health education, serving as a means of achieving sustained outcomes.

**Fig. 2:**
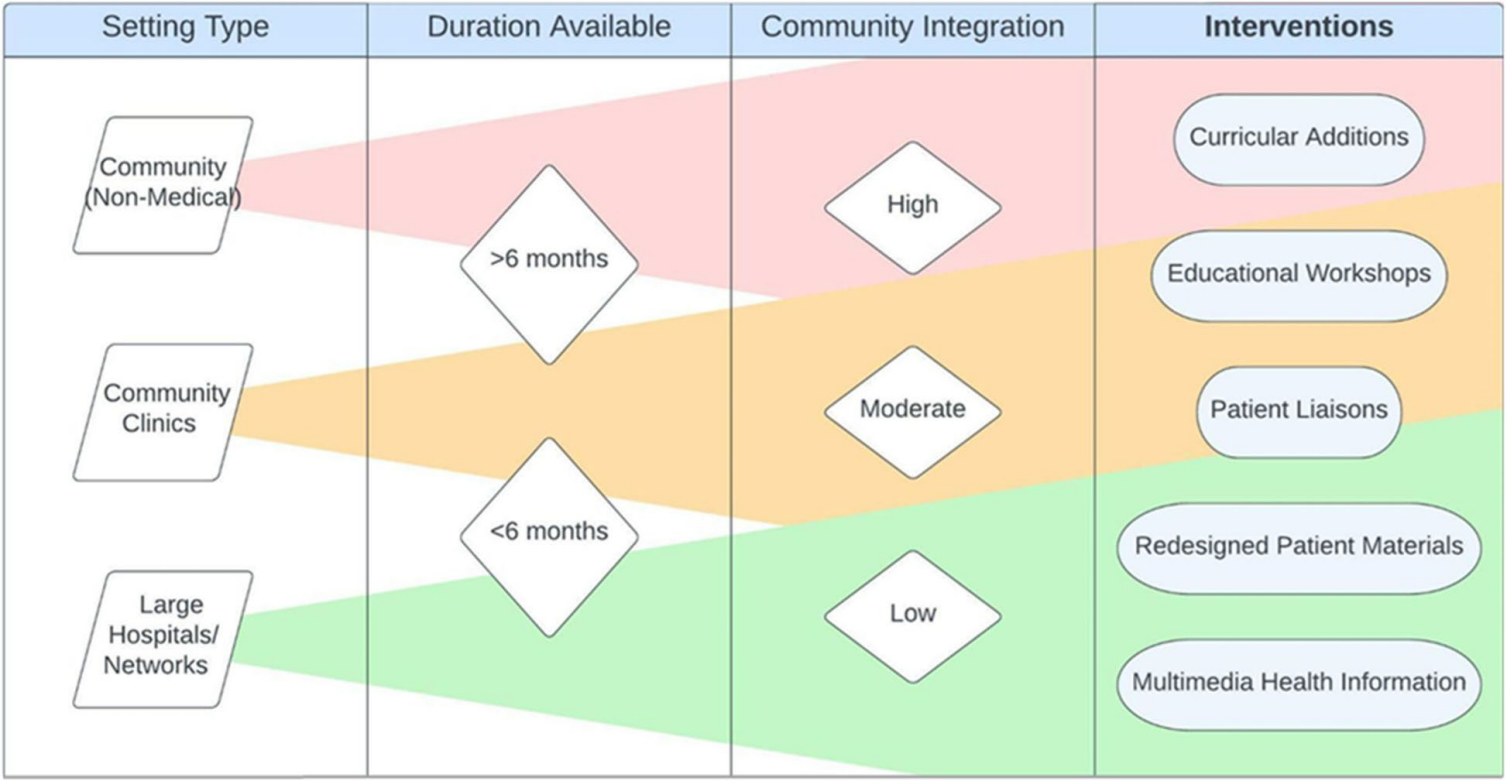
Recommendations for intervention models by common combinations of setting type, duration available, and community engagement

Other elements of successful HL interventions may include finding a fit between factors such as intervention type, size and type of setting, duration of time available, and level of community integration (Fig. 2). As described above, HL may be framed as organizational as well as individual, and successful interventions better integrate organizational setting into the structure of the HL intervention. We refer to community integration as the level of incorporation of community resources, stakeholders such as *promotores*, and settings into interventions aimed at improving health literacy, concepts drawn from the literature [94, 95]. These categories of community integration were inferred from the setting type since we expect large hospitals to be less involved in community initiatives than community clinics or community settings (i.e. local churches) themselves. Smaller, community-based settings and nonmedical centers such as churches and college campuses seemed to be more successful with implementing multiweek curricula interventions. This may be because these settings have the infrastructure in place for recruitment and retention of community members with a SLP. Larger hospital systems and clinics with less time and resources available may be better able to focus on culturally and linguistically concordant patient materials and replace standard of care materials written in English with multimedia health information. These recommendations are illustrated in Fig. 2, which displays fit between intervention type and setting.

The findings of our review are also relevant to studies of HL in other populations with a non-English preference, including minority, migrant or immigrant populations. Previous reviews of HL interventions did not include studies measuring HL indirectly through variables such as health outcomes or behavioral change but only included those using standardized tools [85]. However, as these standardized assessment tools are available predominantly in English, this approach may limit the generalizability of past reviews to non-English speaking populations. A growing body of evidence suggests that a reframing of our understanding of HL, especially among marginalized communities, is necessary to improve health equity [2].

Finally, our review highlights a need for additional attention to the development and adaptation of HL interventions for patients with SLP in the United States. Policies promoting HL interventions may need to better address the needs of specific populations through research and the widespread promotion of effective strategies.

### Limitations

A limitation of our review is that all studies were conducted in the United States, which limits the generalizability of our findings to healthcare systems in other countries. It should be noted that we did not explore gray literature. We also chose to limit our review to studies that took place after 2010, preventing a fuller historical examination of HL literature. Finally, we could not conduct a meta-analysis due to variability in design and measurement.

## Conclusion

There is a small but growing body of literature that addresses the need for HL interventions among individuals in the United States with SLP. However, there is no consensus around strategies to improve or tools to assess HL, and studies vary greatly in quality and risk of bias. Important target populations, such as children, older adults and men, may be excluded from this research. Strategies that incorporate linguistic and cultural factors particular to this population, such as *fotonovelas* and health promoters from similar cultural backgrounds, may be of use in promoting HL. There is a need for improved research and policy on HL interventions specifically targeting this population.

### Supplementary Information

Supplementary Material 1.

Supplementary Material 2.

Supplementary Material 3.

## Data Availability

No datasets were generated or analysed during the current study.

## Abbreviations

HL: Health literacy
SLP: Spanish-language preference
PRISMA: Preferred Reporting Items for Systematic Reviews and Meta-Analysis
RCTs: Randomized control trials
PP: Pre/post
PC: Prospective cohort
CC: Cross-sectional
RoB 2: Revised Tool to Assess Risk of Bias in Randomized Trials
ROBINS -I: Risk Of Bias In Non-randomized Studies - of Interventions
SWiM: Synthesis without Metanalysis
FQHCs: Federally Qualified Health Centers
TOFHLA: Test of Functional Health Literacy in Adults
NVS: Newest Vital Sign
DHL: Diabetes health literacy
SP: Spanish-speaking
ES: English-speaking
TT: Tummy time
UC: Usual Care
IC: Intervention Care
PA: Physical activity
EHS: Early Head Start
KAP: Knowledge, Attitudes, Perception
PED: Pediatric Emergency Department
ECHO: Empowering Change in Health Outcomes
H-SCALE: Hypertension Self-Care Activity Level Effects
ADDIE: Analysis, Design, Develop, Implement, and Evaluate
PaCKS: Palliative Care Knowledge Scale
ESRD: End-stage renal disease
D-Lit: Depression Literacy Questionnaire
DSS: Depression Stigma Scale
ATSPPH-SF: Attitudes Towards Seeking Professional Psychological Help Short Form
EPPM: Extended Parallel Process Model
HPD: Hearing protection devices
PHM: Preventive health model
REALM-SF: Rapid Estimate of Adult Literacy in Medicine—Short Form
PHQ-9: 9-item Patient Health Questionnaire
DKM: Depression Knowledge Measure
VAS: Visual analog scale
PDSA: Plan, Do, Study, Act
MUQ: Medication Understanding Questionnaire
