# Supplementary material for: A systematic review and narrative synthesis of health literacy interventions among Spanish speaking populations in the United States
Source: BMC Public Health. 2024 Jun 27;24:1713. doi: 10.1186/s12889-024-19166-6 (PMC11210103; doi:10.1186/s12889-024-19166-6)
Supplement: Supplementary file 2 — Supplementary Material 2. [file 12889_2024_19166_MOESM2_ESM.docx]

Supplementary Table 1

| Preliminary Review Search Strategy |
| --- |
| PubMed, Embase, Web of science |
| *“health literacy” AND*  *‘intervention’ OR ‘Spanish’, ‘Hispanic’OR ‘LEP’,* OR*‘limited English proficiency’* using the |
| PubMed example: ((limited English proficiency[MeSH Terms]) OR (Hispanic)) OR (Spanish*)) AND (health literacy) AND (intervention). |

| Final Search Stategy | **Limit: 2011 to present** |
| --- | --- |
| **PubMed** | (“limited English proficiency”[mh] OR “limited English proficiency”[tw] OR “English proficiency”[tiab:~3] OR “language barrier”[tw] OR “Hispanic or Latino”[mh] OR Spanish*[tw] OR Hispanic*[tw] OR Latino*[tw] OR  Latina*[tw] OR Latinx[tw] OR Latine[tw] OR “Latin American”[tw] OR “Central American”[tw] OR “South American”[tw] OR “Cuban American”[tw] OR “Mexican American”[tw] OR “Puerto Rican”[tw] OR Chicano*[tw] OR Chicana*[tw])  AND  (“health literacy”[mh] OR “health literacy”[tw] OR “ehealth literacy”[tw])  AND  (education[subheading] OR curriculum[mh] OR radio[mh] OR “graphic novels as topic”[mh] OR “patient education as topic”[mh] OR “health promotion”[mh] OR “health education”[mh] OR “peer group”[mh] OR “cartoons as topic”[mh] OR “patient navigation”[mh] OR pamphlets[mh] OR “video recording”[mh] OR multimedia[mh] OR intervention[tw] OR promotores[tw] OR fotonovela[tw] OR “language concordance”[tw] OR “health education”[tw] OR narration[tw] OR curriculum[tw] OR radio[tw] OR “graphic novella”[tw] OR “lay health advisors”[tw] OR “decision aid”[tw] OR “entertainment education”[tw] OR “peer education”[tiab:~3] OR Navegantes[tw] OR “instructional video”[tw] OR “educational video”[tw] OR “patient navigation”[tw] OR “multimedia kiosk”[tiab:~1] OR “illustrated medication list”[tiab:~1])  AND 2011:3000/12/12[pdat] |
| Embase | ('limited English proficiency'/exp OR ‘limited English proficiency’:ti, ab OR (English NEAR/3 proficiency) OR ‘language barrier’:ti, ab OR 'Hispanic'/exp OR Spanish*:ti, ab OR Hispanic*:ti, ab OR Latino*:ti, ab OR Latina*:ti, ab OR Latinx:ti, ab OR Latine:ti, ab OR ‘Latin American’:ti, ab OR ‘Central American’:ti, ab OR ‘South American’:ti, ab OR ‘Cuban American’:ti, ab OR ‘Mexican American’:ti, ab OR ‘Puerto Rican’:ti, ab OR Chicano*:ti, ab OR Chicana*:ti, ab)  AND  ('health literacy'/exp OR ‘health literacy’:ti, ab OR ‘ehealth literacy’:ti, ab)  AND  ('intervention study'/exp OR 'curriculum'/exp OR 'adult education'/exp OR 'health education'/exp OR 'health promotion'/exp OR 'patient education'/exp OR 'videorecording'/exp OR 'lay health worker'/exp OR intervention:ti, ab OR promotores:ti, ab OR fotonovela:ti, ab OR ‘language concordance’:ti, ab OR ‘health education’:ti, ab OR narration:ti, ab OR curriculum:ti, ab OR radio:ti, ab OR ‘graphic novella’:ti, ab OR ‘lay health advisors’:ti, ab OR ‘decision aid’:ti, ab OR ‘entertainment education’:ti, ab OR (peer NEAR/3 education) OR Navegantes:ti, ab OR ‘instructional video’:ti, ab OR ‘educational video’:ti, ab OR ‘patient navigation’:ti, ab OR ‘multimedia kiosk’:ti, ab OR ‘illustrated medication list’:ti, ab)  AND (2011:py OR 2012:py OR 2013:py OR 2014:py OR 2015:py OR 2016:py OR 2017:py OR 2018:py OR 2019:py OR 2020:py OR 2021:py OR 2022:py OR 2023:py)  AND ‘article’/it |
| **Web of Science** | (“limited English proficiency” OR (English NEAR/3 proficiency) OR “language barrier” OR Spanish* OR Hispanic* OR Latino* OR Latina* OR Latinx OR Latine OR “Latin American” OR “Central American” OR “South American” OR “Cuban American” OR “Mexican American” OR “Puerto Rican” OR Chicano* OR Chicana*)  AND  (“health literacy” OR “ehealth literacy”)  AND  (intervention* OR promotores OR fotonovela OR “language concordance” OR “health education” OR narration OR curriculum OR radio OR “graphic novella” OR “lay health advisor” OR “decision aid” OR “entertainment education” OR (peer NEAR/3 education) OR Navegantes OR “instructional video” OR “educational video” OR “patient navigation” OR “multimedia kiosk” OR “illustrated medication list”) |
