# Supplementary material for: A systematic review and narrative synthesis of health literacy interventions among Spanish speaking populations in the United States
Source: BMC Public Health. 2024 Jun 27;24:1713. doi: 10.1186/s12889-024-19166-6 (PMC11210103; doi:10.1186/s12889-024-19166-6)
Supplement: Supplementary file 3 — Supplementary Material 3. [file 12889_2024_19166_MOESM3_ESM.docx]

Appendix 1

| **Reviewer Name** |
| --- |
| **Title** |
| **Author** |
| **Journal** |
| **Study Design** |
| **Summary** |
| **Aim (HL subject addressed, i.e., diabetes, hypertension, etc.)** |
| **Setting & Region (i.e., HIV community-based clinic (Los Angeles))** |
| **Target population (i.e., children in grades 4 and 5)** |
| **Number of participants/Demographic info (Education level, country of origin, and fluency in english)** |
| **Intervention** |
| **Professionals (i.e., Health Educators: Nurse Supervisors: Social Workers Director of Psychosocial Services: Program and Clinical Trial Coordinator)** |
| **Comparator/control group (if applicable)** |
| **HL Assesment used & Scores** |
| **Desired outcome and how it was measured** |
| **Results/outcome data** |
| **Secondary outcomes (w/results)** |
| **Covariate Analysis (if applicable)** |
| **Limitations** |
| **Conclusions/Future Directions** |
